# Gastroenteritis Outbreak in British Troops, Iraq

**DOI:** 10.3201/eid1110.050298

**Published:** 2005-10

**Authors:** Mark S. Bailey, Christopher J. Boos, Guy Vautier, Andrew D. Green, Hazel Appleton, Chris I. Gallimore, Jim J. Gray, Nicholas J. Beeching

**Affiliations:** * Army Medical Directorate, Camberley, United Kingdom; † Ministry of Defence, London, United Kingdom; ‡ Health Protection Agency, London, United Kingdom; § Liverpool School of Tropical Medicine, Liverpool, United Kingdom

**Keywords:** Gastroenteritis, military personnel, Iraq, calicivirus infections, Norovirus, Sapovirus, hospitals, military, cross infection, infection control, environmental health, dispatch

## Abstract

Gastroenteritis affected many British military personnel during the war in Iraq. In the first month, 1,340 cases were seen; 73% of patients required hospital admission and 36% were hospital staff. In a survey of 500 hospital staff, 76% reported gastroenteritis, which was more likely in clinical workers. Investigations showed only caliciviruses.

Infectious diseases are a frequent problem in military campaigns and usually cause more casualties than battlefield injuries (*1*). Even when they do not have a high death rate, the illness caused may still diminish operational effectiveness. Military deployments are often affected by gastroenteritis (*2**,**3*), in particular viral gastroenteritis (*4*). Norovirus gastroenteritis affected troops during both the Gulf War in 1991 (*5*) and the Afghanistan campaign in 2002 (*6*). Caliciviruses (which include both noroviruses and sapoviruses) are well adapted to cause such outbreaks because of their high infectivity, multiple routes of transmission, presymptomatic and postsymptomatic viral shedding, high survivability in the environment, resistance to disinfectants, and poor long-term immunity after infection. The effects and economic costs of these infections in hospital facilities have recently been assessed (*7*).

British troops invaded Iraq on March 21, 2003, and lived in minimal hygiene facilities for several weeks. During the first week of the conflict, they ate individual ration packs only, but centralized catering and locally produced fresh rations (including salads and fruit) were introduced during the second week. A 200-bed British military field hospital was established in Iraq on March 26 for 2 months. During this time 26,000 British troops were in the area, and ≈50% were close enough to visit the hospital with illnesses such as gastroenteritis. A large outbreak of gastroenteritis affected many British units, including the staff of the field hospital. We reviewed the outbreak in patients from local units and assessed its effect on the field hospital.

## The Study

Data from hospital records were collected from March 28, 2003, when the first gastroenteritis cases were observed, until April 29, 2003, when the number of gastroenteritis cases decreased to <20 per day. A subsequent retrospective survey of hospital staff was carried out from April 19 to May 3, 2003, when the number of cases began to decrease. The survey used a face-to-face structured questionnaire designed to study possible risk factors and outcomes associated with gastroenteritis. Gastroenteritis was defined as having >2 of the following: diarrhea, abdominal pain, nausea, vomiting, and either myalgia, headache, or subjective fever. The presence of any of the last 3 symptoms was counted as 1 symptom overall.

Because of the situation and number of cases, conducting fecal investigations for all patients was not possible. Instead, investigations were targeted at the most severe or atypical cases after clinical assessment on daily postadmission ward rounds. Fecal samples were collected within 24 hours of admission. Ova, cysts, and parasites were sought by microscopy with standard wet-preparation techniques and staining with iodine where indicated. Enteropathic bacteria, including *Campylobacter* spp., *Salmonella* spp., *Shigella* spp., and *Escherichia coli* O157, were detected by standard microbiologic culture techniques. We tested for *Vibrio* spp. where clinically indicated. Eight refrigerated fecal samples were sent to the United Kingdom reference laboratory for enteric viruses (Centre for Infections, Health Protection Agency), where enteropathic viruses were sought by electron microscopy and reverse transcription–polymerase chain reaction (RT-PCR) (*8*). These studies were reviewed and approved by the field hospital ethics committee.

Statistical analysis of results was carried out by using SPSS version 12 software (SPSS Inc., Chicago, IL, USA). Unadjusted odds ratios (ORs) were derived from 2 × 2 tables. Unadjusted ORs for age groups were determined by using a chi-square test for linear trend. Adjusted ORs (AORs) were estimated by logistic regression analysis by using a backwards elimination technique. Variables with the lowest significance were sequentially removed until the best fit model was achieved.

**Figure 1:**
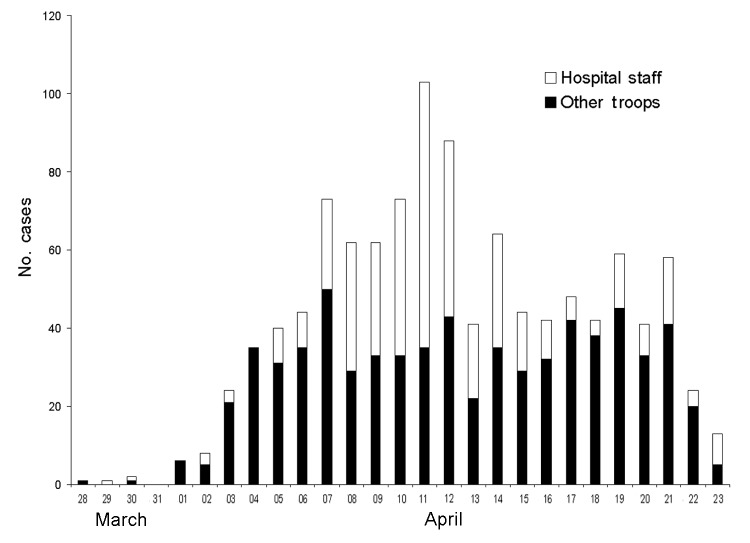
Gastroenteritis patients who came to the field hospital in March and April 2003.

During the first month of the conflict, 2,065 patients were seen at the hospital, of whom 1,466 (71%) were managed by internal medicine physicians. Among these, 1,340 (91%) had gastroenteritis; 975 (73%) required admission. The epidemic distribution of gastroenteritis patients who came to the hospital is shown in Figure 1. Admission rates were initially 93% but gradually decreased to 13% during the study period. Mean length of stay in the hospital was 1.65 days, which resulted in 1,608 bed-days occupied, and bed occupancy rates reached >90%. Hospital staff accounted for 36% of all gastroenteritis patients seen. Medical units and armored units appeared to be most affected.

Five hundred (77%) of 648 hospital staff were surveyed when the number of cases began to decrease. Among these, 382 (76%) had gastroenteritis, of whom 202 (53%) had diarrhea that persisted for >48 hours longer than their other symptoms (range 2–14 days). Overall, 292 (60%) of the hospital staff surveyed visited the hospital for medical care, and 192 (39%) were admitted because of gastroenteritis. Those affected required a mean (SD) of 2.1 (1.7) days off work; 796 work days were lost in the 77% of staff surveyed.

Among the 382 hospital staff with gastroenteritis, symptoms are shown in Table 1, and risk factors for gastroenteritis compared with unaffected staff are shown in Table 2. Gastroenteritis was more likely in clinical staff (AOR 1.98, 95% confidence interval [CI] 1.28–3.01) and women (AOR 1.63, 95% CI 1.00–2.65), but no association was found between gastroenteritis and age, blood group, or reported contamination of living areas by vomit, feces, or both.

**Table 1:** Symptoms in 382 hospital staff with gastroenteritis

Symptom	No. (%)
Diarrhea	341 (89)
Abdominal pain	323 (85)
Nausea	304 (80)
Vomiting	215 (56)
Fever (subjective)	212 (55)
Myalgia	210 (55)
Headache	209 (55)

**Table 2:** Analysis of risk factors for gastroenteritis in 500 hospital staff*

Risk factor	GE, no. (%) (n = 382)	No GE, no. (%) (n = 118)	OR (95% CI)	p value	AOR (95% CI)
Clinical worker	208 (54)	43 (36)	2.09 (1.36–3.19)	<0.01	1.98 (1.28–3.01)
Female	153 (40)	35 (30)	1.58 (1.02–2.47)	<0.05	1.63 (1.00–2.65)
Contaminated accommodation	138 (36)	53 (45)	0.69 (0.46–1.05)	0.09	–
Age, y					
18–29	126 (33)	36 (31)	1.00 (Referent)	–	–
30–39	155 (41)	51 (43)	0.87 (0.52–1.45)	0.57	–
>39	101 (26)	31 (26)	0.93 (0.52–1.67)	0.80	–
Blood group					
O	181 (47)	54 (46)	1.07 (0.69–1.65)	0.76	–
A	147 (38)	53 (45)	0.77 (0.49–1.19)	0.21	–
B	36 (9)	7 (6)	1.65 (0.68–4.19)	0.24	–
AB	18 (5)	4 (3)	1.41 (0.44–5.03)	0.54	–

*GE, gastroenteritis; OR odds ratio; CI, confidence interval; AOR, adjusted odds ratio after logistic regression analysis.

**Figure 2:**
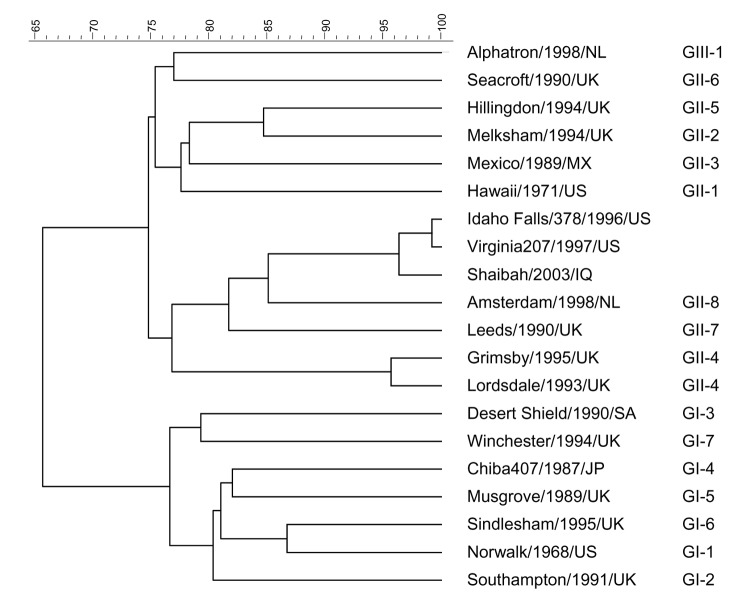
Dendrogram of the 5´ end of open reading frame 2 of noroviruses, including the Shaibah strain. Scale at the top shows percent relatedness between different strains. GenBank strains are Alphatron/1998/NL (AF195847), Seacroft/1990/UK (AJ277620), Hillingdon/1994/UK (AJ277607), Melksham/1995/UK (X81879), Mexico/1989/MX (U22498), Hawaii/1972/US (U07611), Idaho Falls/378/1996/US (AY054299), Virginia207/1997/US (AY038599), Amsterdam/1998/NL (AF195848), Leeds/1990/UK (AJ277608), Grimsby/1995/UK (AJ004864), Lordsdale/1995/UK (X86557), Desert Shield/1990/SA (U04468), Winchester/1995/UK (AJ277609), Chiba407/1987/JP (AB022679), Musgrove/1989/UK (AJ277614), Sindlesham/1995/UK (AJ277615), Norwalk/1969/US (M87661), and Southampton/1991/UK (L07418). The sequence of the Shaibah/2003/IQ strain can be obtained from the Enteric Virus Unit, Virus Reference Department, Centre for Infections, Health Protection Agency (christopher.gallimore@hpa.org.uk).

Fecal microscopy and culture were conducted for 69 patients during this 2-month deployment. No parasites or bacterial pathogens were found during the first month when the initial outbreak occurred. Only 2 fecal samples (collected during week 2 of the outbreak) arrived at the UK reference laboratory. Both samples showed caliciviruses by electron microscopy and had the classic appearance usually associated with sapoviruses rather than noroviruses. They could not be identified by RT-PCRs for noroviruses, sapoviruses, or astroviruses. However, using norovirus primers that amplified contiguous regions of open reading frames (ORFs) 1 and 2 (*9*), we detected a norovirus-specific amplicon in 1 sample. Subsequently, the norovirus was identified by cDNA sequencing as an unusual strain with 96% identity to NV/Idaho Falls/378/1996/US (GenBank accession no. AY054299) and designated NV/Shaibah/2003/IQ (Figure 2). This strain does not group within any of the 15 previously identified norovirus genotypes (*10*) but is similar to novel strains identified in US military personnel during this same period (*11*).

## Conclusions

This study was conducted in a war zone by clinicians in a busy military field hospital. Surveying hospital cases inevitably underestimates the true scale of the problem faced by military units in Iraq. The fact that 71% of patients were seen by internal medicine physicians is typical of the disease-to-trauma ratio that occurs in military conflicts (*1*) and overseas training exercises (*12*).

Gastroenteritis was the major cause of illness in hospital cases during this operation and probably diminished the operational effectiveness of those units affected. Admission rates were initially high because of exhaustion, dehydration, and poor living conditions during the first month of the conflict. Viral gastroenteritis is readily transmitted in healthcare settings (*7*) and confined living spaces, which may explain why medical and armored units were most affected. Environmental health measures planned for this deployment had high standards, but they were not fully implemented in time to prevent this outbreak, which coincided with the delivery of fresh rations.

The survey of hospital staff indicates the scale of the problem faced by the field hospital, but this may also be an underestimate since cases were still occurring while data were collected. Since gastroenteritis was more common in clinical workers, infection control at the hospital may have been inadequate. Hygiene standards for the hospital were also high (*13*), but they were not fully implemented in time to prevent the outbreak among hospital staff.

The wide epidemic distribution suggests a prolonged period of disease transmission, rather than a single point source outbreak. Although this outbreak fulfills the criteria of Kaplan et al., which suggest that a calicivirus was responsible (*14*), data were insufficient to determine the cause. However, a similar outbreak on a Royal Navy hospital ship occurred during this period when locally produced fresh salad was taken on board; several enteric viruses were subsequently identified (*8*).

This outbreak in Iraq would have had an even greater effect if it had been caused by a virulent species of *Salmonella* or *Shigella*, which are common foodborne pathogens in this region. We believe introducing fresh rations during a military operation, before adequate health and infection control measures were fully implemented, was inadvisable. In contrast, Royal Marines based on the Al-Faw Peninsula were not supplied with fresh rations during this period, and no outbreaks of gastroenteritis were reported (S. Bree, pers. comm.). More rigorous, prospective studies are required to understand the epidemiology and effect of gastroenteritis in deployed military personnel (*15*).

We recommend that improvements be made in the implementation of environmental health and infection control measures during operational deployments. Military units should avoid fresh rations during military operations until adequate hygiene measures have been fully implemented and inspected. Further studies and changes to working practices are required to prevent and control similar outbreaks in the future.
